# Supplementary material for: Comparing the impact of genotypic based diagnostic algorithm on time to treatment initiation and treatment outcomes among drug-resistant tuberculosis patients in Amhara region, Ethiopia
Source: PLoS One. 2021 Feb 18;16(2):e0246938. doi: 10.1371/journal.pone.0246938 (PMC7891731; doi:10.1371/journal.pone.0246938)
Supplement: Attachment — Submitted filename: Review PONE-D-20-18404.docx [file pone.0246938.s001.docx]

**Review:** **‘Comparing the impact of Genotypic Based Diagnostic Algorithm on Time to Treatment Initiation and Treatment Outcomes among Drug-Resistant Tuberculosis Patients in Amhara Region, Ethiopia’.**

**PONE-D-20-18404**

The topic under discussion is very relevant and important to understand the effect of the molecular diagnostics in the early diagnosis and treatment initiation, and its impact on patient-level treatment outcome. In the present form, the manuscript needs major revision and correction in its methodology and analysis.

**Major comments:**

**Abstract**

The methods and the result section in the abstract need to be revised based on the detailed comments in the methods section. The major comments are around median time to treatment and categorization of the three comparison groups and treatment outcomes.

Line 41, ’in smear-positive patients’, your discussion shows the opposite, and your data does not fully support this. Please revise.

Line 30, 32, 35, 39 and 42, **GeneXpert** is the instrument (or the platform) and **Xpert MTB/RIF** is the assay (test), please correct this throughout the manuscript.

**Introduction**

Line 58-63: your paper is on drug-resistant TB, the references you used from 6 to 9 are on drug-sensitive TB and thus do not apply to your study. Please replace them with the relevant references.

Line 72-72, sentence starting ‘As compared to culture….’. The sentence is not correct, the overall sensitivity of Xpert never been that high. If you meant, among smear-positive that might be possible. If so, qualify the sentence accordingly.

Line 80 and 82, reference number 6 is on drug-sensitive TB and thus does not apply to your study. Please replace it as appropriate

**Methods**

In the methods part, the following are missing:

1. Since the three tests are usually conducted one after another, it is not clear how the three distinctive comparison groups were categorized. For instance, if a sample tested positive by Xpert MTB/RIF with Rifampicin Resistance, then LPA needs to be conducted to confirm for Isoniazid resistance. Please clarify, how the three exclusive comparison groups (Xpert MTB/RIF, LPA, and Culture) were created, whether the country was using three different algorithms at different times?
2. The definition of terms for treatment outcomes is not appearing in the methods part. Also, the categorization of treatment outcome needs to be reconsidered. A comparison of outcome is helpful when categorized as successful (cured and treatment completion) and unsuccessful (failure, died, and lost to follow-up). *You may use references Johnston et al PLOS ONE 2009 or Evan* *[W Orenstein BS](https://www.sciencedirect.com/science/article/abs/pii/S1473309909700416#!) et al, the Lancet infectious disease, 2009).* Since we don’t know the final treatment outcome, transferred out needs to be excluded when you compare treatment outcome. Please revise Table 4.
3. The measurement of the median time, particularly the ‘time to treatment initiation’ is not clear. It is not immediately clear how do you define a diagnosis, and thus time to diagnosis? Please clarify and include in your methods. Time from the first visit to diagnosis and time from diagnosis to treatment are subsets of time from the first visit to treatment. Based on this, the median time from initial visit to treatment does not seem to right or at least the numbers do not make sense in Table 3. Please clarify how time to treatment is less than that of time to diagnosis and time to treatment (from diagnosis).
4. MDR-TB treatment regimens are one of the major factors affecting treatment outcomes, and there is no mention of MDR-TB treatment regimens in the study period (2010-2017). The WHO changed the recommendation to drug-resistant treatment in 2011 and 2016. Also, if there was a change in treatment regimen between 2010 and 2017 it needs to be clarified.
5. Line 100-101, the sentence starting ‘over 90% of DR_TB….’ needs a reference.
6. Line 123, it is not immediately clear why the smear result was an excursion criterion. The diagnosis of drug-resistant TB is not based on the smear. Please clarify if you still want to keep it as exclusion criterion.
7. You need to include another table indicating the distribution of patients among the four hospitals, and which diagnostic tool used in which hospital.
8. You need to include which culture method was used, solid culture versus liquid culture.

**Results**

1. Table 1, the median age in years…the 16.75, 28.5 and 17 are not IQR, this needs correction
2. Table 1, ‘smear result not recorded’ should be taken out based on your exclusion criteria
3. Tables 2 and 3 need revision based on the comments on time to treatment in the methods.
4. Line 152, since the 34 don’t have recorded results, based on your exclusion criteria 14 should be 48 (14 plus 34).
5. Line 181, you did well by excluding the 34. That is the same reason you should exclude the 34 throughout.
6. Line 194, ‘six patients were on both’…. this is not clear when you state both treatments, which is which?
7. Table 4, need revision based on the recommended treatment outcome categorization (successful versus unsuccessful treatment outcome). See the previous comment in the methods section.

**Discussions**

Line 229-231, the sentence starting with ‘the rationale for this….’ This does not sound true. In fact, the opposite is true. The lower bacteria load delays time to culture positivity and the DST that follows culture. Please clarify or correct.

Line 232-237, this discussion is likely to change based on the comments in the methods and result on treatment outcomes (Table 4). Please revise it accordingly.

Limitations: line 240, why was ‘the health care facilities’ included as missing data. Didn’t you know the four hospitals you studied? In fact, would have been best if you included the result on the comparison between the hospitals in median times and treatment outcomes. Also, the distribution of patients among the four hospitals is missing. Wiser if you include those. As a limitation, you also need to include the lack of data on socioeconomic status and mode of treatment whether DOTS, at facilities or community based, as some of the factors potentially affecting treatment outcomes.

**Conclusions and recommendation**

Line 247…’particularly in smear-positive patients’, your discussion shows the opposite, and your data does not fully support this. Please revise.

Line 247-248 may change after your reanalysis of treatment outcomes (Table 4). Please revise it accordingly.

**Minor comments:**

**Abstract**

Line 27 and 28, delete ‘with p-value’ and ‘finally, the p-value was reported’. Mentioning these may not be that necessary.

**Introduction**

Line 54 and 55, tuberculosis is already abbreviated, thus use it as TB moving forward. There are lots of places in the manuscript like this, please correct accordingly.

Line 57, LPA need to be written in full since first-time use.

Line 58, replace the sentence with…. ‘Shorten time to diagnosis, and reduced delay to treatment among DR-TB, and improved final treatment outcomes.’

Line 62, **GeneXpert** is the instrument (or the platform) and **Xpert MTB/RIF** is the assay (test), please correct this throughout the manuscript.

Line 90, replace ‘we are intended’ with ‘we aim’

**Methods**

Line 105, add a comma after historically,

Line 116, replace the sentence in parenthesis with ‘data difference from the diagnosis to initiation of treatment.’

**Result**

Line 154, replace ‘three-hundred twenty-nine’ with 329

Line 155, remove ‘with’ after 28 and add old after ‘years’

Line 160, replace ‘drug-resistant tuberculosis’ with DR-TB.

Line 161, replace ‘DR-TB diagnosis’ with ‘diagnostic’

Line 164-167, the use of semi-colon and comma does not flow well. Replace the sentences with *‘The overall median time and IQR in days from first care-seeking visit to diagnosis, from diagnosis to treatment, and from first care-seeking to treatment were: 2 (0,9), 3 (1,8) and 4 (1,11) for Xpert MTB/RIF; 4 (1,55), 33 (4,76) and 3 (1,12) for LPA; and 70 (18,182), 44 (9, 145) and 76 (3.75,191) for culture.’*  …your numbers of course will be corrected per the revised analysis and result.

Line 167, remove respectively.

Line 194, use a text for a number in a single digit. Replace 6 with ‘six’

Line 194, add ‘s’ after the patient (patients) and replace was with were. Six patients were…..

**Discussion**

Line 212, replace ‘diagnosis TD-TB tool’ with ‘diagnostic tool’

Line 216, replace GeneXpert with ‘Xpert MTB/RIF’

Line 216, Xpert MTB/RIF test is not simple. It is a very complex process, but easy to perform. Thus replace ‘a simple diagnostic assay’ with ‘an easy to do diagnostic assay’.

Line 239, add ‘data’ after missing.

**Acknowledgment**

Line 253, remove ‘s’ from datas…

**Authors’ contribution**

Replace ‘Dta’ with ‘Data’.

**References**

Line 343, Ref # 24, seems not well written. Please revise.

Line 363-4, the reference seems missing the publication number. Please revise.
