# Supplementary material for: Comparing the impact of genotypic based diagnostic algorithm on time to treatment initiation and treatment outcomes among drug-resistant tuberculosis patients in Amhara region, Ethiopia
Source: PLoS One. 2021 Feb 18;16(2):e0246938. doi: 10.1371/journal.pone.0246938 (PMC7891731; doi:10.1371/journal.pone.0246938)
Supplement: Attachment — Submitted filename: Response to Reviewers.docx [file pone.0246938.s002.docx]

**Date August/25/2020**

**Rebuttal letter**

**PONE-D-20-18404**

**Comparing the impact of Genotypic Based Diagnostic Algorithm on Time to Treatment Initiation and Treatment Outcomes among Drug-Resistant Tuberculosis Patients in Amhara Region, Ethiopia**

**Mehari Woldemariam Merid**

**To PLOS ONE**

Dear all,

We are very pleased to thank you for providing very relevant, constructive, and critical comments and questions about the manuscript. Based on your comments and questions, we authors have tried to review the document in detail. The point by point responses are presented in two columns as reviewers’ comments and authors’ response as follows. The detail revision and changes we made in the main document is described as track change in the document attached separately. We hope the revised manuscript will better suit the journal, but we are happy to consider farther revisions.

| **Reviewers comment** | **Authors response** |
| --- | --- |
| **Major comments:** |  |
| **Abstract** |  |
| The methods and the result section in the abstract need to be revised based on the detailed comments in the methods section. The major comments are around median time to treatment and categorization of the three comparison groups and treatment outcomes. | **Answer: Dear Reviewer, thank you very much for your fruitful comment.**  According to your comment, we have corrected the questions you have raised and incorporated on the main documents. We have also made clarifications or re-write on the topics which need re-writings or clarifications. |
| Line 41, ’in smear-positive patients’, your discussion shows the opposite, and your data does not fully support this. Please revise. | **Answer: Thank you, dear reviewer.**  Dears, this conclusion was made based on our result as indicated in **Table 3**, which shows Xpert MTB/RIF significantly reduced the median time from first care-seeking visit to diagnosis, diagnosis to treatment initiation, and first care-seeking visit to treatment initiation among smear-positive DR-TB as compared to the other diagnostic methods. The problem here might be the way we have discussed, now we have tried to discuss it clearly in the revised manuscript. **Page: 15 &16, Line: 222-241** |
| Line 30, 32, 35, 39 and 42, **GeneXpert** is the instrument (or the platform) and **Xpert MTB/RIF** is the assay (test), please correct this throughout the manuscript. | **Answer: Thank you, reviewer, for your comment.**  Agreeing to your comment we have corrected it throughout the entire document. |
| **Introduction** |  |
| Line 58-63: your paper is on drug-resistant TB, the references you used from 6 to 9 are on drug-sensitive TB and thus do not apply to your study. Please replace them with the relevant references. | **Answer: Thank you, dear reviewer, for the comment.**  Accordingly, we have agreed with your comment. Currently, we have corrected and replaced with references done on DR-TB in the main document. **Page: 4, Line: 57, 59 & 60** |
| Line 72-72, sentence starting ‘As compared to culture….’. The sentence is not correct, the overall sensitivity of Xpert never been that high. If you meant, among smear-positive that might be possible. If so, qualify the sentence accordingly. | **Answer: Thank you, dear reviewer, for the comment.**  Dear reviewer, we have re-read the paper and they have done on presumptive TB patients not among smear-positive patients. They have also compared the Xpert MTB/RIF test with the MGIT culture test and reported as the Xpert MTB/RIF had 99.1% sensitivity and 96% specificity to detect DR-TB cases compared to MGIT culture. We have also read and tried to find the reasons for this finding in their discussion part, but they didn’t put any further justifications. |
| Line 80 and 82, reference number 6 is on drug-sensitive TB and thus does not apply to your study. Please replace it as appropriate | **Answer: Thank you, dear reviewer.**  Dear reviewer, now we have corrected it according to your recommendations on the main document.  **Page: 5, Line: 75 & 77** |
| **Methods**  In the methods part, the following are missing: |  |
| 1. Since the three tests are usually conducted one after another, it is not clear how the three distinctive comparison groups were categorized. For instance, if a sample tested positive by Xpert MTB/RIF with Rifampicin Resistance, then LPA needs to be conducted to confirm for Isoniazid resistance. Please clarify, how the three exclusive comparison groups (Xpert MTB/RIF, LPA, and Culture) were created, whether the country was using three different algorithms at different times? | **Answer: Thank you, dear reviewer, for raising this concern.**  Sure! We have to share your concerns. Xpert MTB/RIF was introduced in the Amhara region, Ethiopia by September 2013, before that culture and LPA was used alternatively to diagnose DR-TB. Even after the introduction of Xpert MTB/RIF in 2013, culture and LPA were also used to diagnose DR-TB because of the limited Xpert MTB/RIF test access. Currently, as you have stated these three tests are done one after the other. Once the patient becomes resistant to Rifampicin by Xpert MTB/RIF then first and second line LPA was performed to see further resistance for INH, FLQ, and injectable. The culture was done to monitor the treatment response and for further phenotypic DST performance. |
| 2. The definition of terms for treatment outcomes is not appearing in the methods part. Also, the categorization of treatment outcome needs to be reconsidered. A comparison of outcome is helpful when categorized as successful (cured and treatment completion) and unsuccessful (failure, died, and lost to follow-up). *You may use references Johnston et al PLOS ONE 2009 or Evan* *[W Orenstein BS](https://www.sciencedirect.com/science/article/abs/pii/S1473309909700416#!) et al, the Lancet infectious disease, 2009).* Since we don’t know the final treatment outcome, transferred out needs to be excluded when you compare treatment outcome. Please revise Table 4. | **Answer: Dear Reviewer, thank you very much for your suggestion.**  Accordingly, we have included the definition of treatment outcome and re-categorized as successful (cured and treatment completion) and unsuccessful (failure, died, and lost to follow-up) and cited the recommended reference as per your suggestion in the method part of the main document. Based on this definition of treatment outcome we have also re-analyzed and revised **Table 4** on the main manuscript.  **Page: 7, Line: 115-119** |
| 3. The measurement of the median time, particularly the ‘time to treatment initiation’ is not clear. It is not immediately clear how do you define a diagnosis, and thus time to diagnosis? Please clarify and include in your methods. Time from the first visit to diagnosis and time from diagnosis to treatment are subsets of time from the first visit to treatment. Based on this, the median time from initial visit to treatment does not seem to right or at least the numbers do not make sense in Table 3. Please clarify how time to treatment is less than that of time to diagnosis and time to treatment (from diagnosis). | **Answer: Thank you, dear reviewer, for inquiring a clarity on these critical issues.**  Accordingly, we clarified that point in the main document as follows. Time from a first care-seeking visit to diagnosis was defined as the total date taken from the patient visit the health facility for care to the time of their tuberculosis diagnosis. Whereas time from diagnosis to treatment initiation was calculated from the date taken from DR-TB diagnosis to anti-TB treatment initiation; and time from first care-seeking visit to treatment initiation was labeled as the date from the patient comes to the health facility for seeking care to the date the patient start anti-TB treatment. **Page: 7, Line: 110-115.**  Dears, we have seen and recognized that the median time from the first care-seeking visit to treatment was lower than the median time from diagnosis to treatment in the LPA method in Table 3 when we analyze and write the result initially. Again, we have critically examined the data one by one based on your comment and we assure you that these findings were true. The reason why this happened was, the data from diagnosis to treatment (IQR) was negatively skewed while the data from the first care-seeking visit to diagnosis and a first care-seeking visit to treatment was positively skewed. As you know from the basic statistics, the median time was taken at the midpoint of all observation. If the data is negatively skewed the median will be higher but if it is a positively skewed data the median will be lower. Hence, the data nature stated here will explain the difference. |
| 4. MDR-TB treatment regimens are one of the major factors affecting treatment outcomes, and there is no mention of MDR-TB treatment regimens in the study period (2010-2017). The WHO changed the recommendation to drug-resistant treatment in 2011 and 2016. Also, if there was a change in treatment regimen between 2010 and 2017 it needs to be clarified. | **Answer: Thank you, Dear reviewer.**  Dears, Of course, the used MDR-TB treatment regimen has a great role on the treatment outcomes of patients. Similarly to the WHO, Ethiopia changed the recommended MDR-TB treatment in 2014, 2018, and 2019. During the study period, the treatment regimen of MDR-TB were changed at once in 2014. Even, in the same guideline, there were two or more recommended regimens for MDR-TB treatment based on the patients' clinical and laboratory parameters. Dear reviewer, there are a bunch of publishing reports on the predictors of final treatment outcomes of MDR-TB in the study area including the used MDR-TB treatment regimens. Plus to this, our objectives were aimed to determine the median time on the diagnosis, treatment initiation, and identify their effect on treatment outcome of different DR-TB diagnosis test. So, the question you have will be appropriate if our study was aimed to identify the factors of MDR-TB treatment outcome. Because of the above mentioned two reasons (it is out of our objectives and the presence of published reports), we differed to see the effect of MDR-TB treatment regimens in our study. |
| 5. Line 100-101, the sentence starting ‘over 90% of DR_TB….’ needs a reference. | **Answer: Thank you, Dear reviewer.**  Now we have cited a reference in the main document. **Page: 6, Line: 96** |
| 6. Line 123, it is not immediately clear why the smear result was an excursion criterion. The diagnosis of drug-resistant TB is not based on the smear. Please clarify if you still want to keep it as exclusion criterion. | **Answer: Thank you, dear reviewer, for your clarity concern.**  The exclusion criteria we have used in this study were those patients diagnose based on clinical criteria. We use sputum smear result as the exclusion criteria while we were comparing the different median time among patients who has sputum smear result and when we have to try to see the effect of the diagnosis test on the final treatment outcome stratified by their sputum smear results. To make it more clear now we have re-write in the main document as follows. “All DR-TB patients diagnosed by Xpert MTB/RIF, LPA, and culture-based DST were included into the study while patients diagnosed on the clinical basis were excluded. To evaluate the effect of diagnosis tests on the final treatment outcome stratified by their sputum smear result, we had excluded those patients who were on treatment and transferred out.” Moreover, now it is presented in **Figure 2** to make it more clearly in this regard. |
| 7. You need to include another table indicating the distribution of patients among the four hospitals, and which diagnostic tool used in which hospital. | **Answer: Thank you, reviewer, for your comment.**  We have already included the number of patients and the used diagnostic methods in each hospital in **Table 1** as the variable name “**Treatment initiating and follow-up site hospital”**. **Table 1,** **Page: 10** |
| 8. You need to include which culture method was used, solid culture versus liquid culture. | **Answer: Thank you, dear reviewer, for your critical comment.**  The solid (Löwenstien-Jensen or LJ) culture media were the used culture method in this study and we have corrected it in the main documents. |
| **Results** |  |
| 1. Table 1, the median age in years…the 16.75, 28.5 and 17 are not IQR, this needs correction | **Answer: Thank you, reviewer, for your critical comment.**  Dears, we apologized for the fault we made, we erroneously inserted the SD instead of the IQR. Now, we have corrected and incorporated it into the main document. **Table 2, Page: 11** |
| 2. Table 1, ‘smear result not recorded’ should be taken out based on your exclusion criteria | **Answer: Thank you, reviewer, for the comment.**  Dear reviewer, Table 1 was presented to show the descriptive of patients’ sociodemographic and clinical characteristics. We didn’t use sputum smear result for exclusion at the outset. Because of this, we have presented the sputum smear status of all the study patients. We excluded those patients who didn’t have smear results when we analyze the data to see the presence of median time and outcome difference among smear-positive and negative patients. To make clearer on the exclusion and inclusion criteria now we have included a diagram in the main document. Please refer to **Figure 2**. |
| 3. Tables 2 and 3 need revision based on the comments on time to treatment in the methods. | **Answer: Thank you, dear reviewer, for the comment.**  Dears, we have addressed this question in question number three in the method part. **Figure 2.** |
| 4. Line 152, since the 34 don’t have recorded results, based on your exclusion criteria 14 should be 48 (14 plus 34). | **Answer: Thank you again, reviewer.**  Dears, to make clear on the inclusion and exclusion criteria, we have re-write and clarified the main documents and **Figure 2.** |
| 5. Line 181, you did well by excluding the 34. That is the same reason you should exclude the 34 throughout. | **Answer: Dear reviewer, thank you very much.**  We intended to compare the impact of diagnostic tests on the final treatment outcomes of DR-TB patients based on their baseline sputum smear status. To do so, 34 patient sputum smear result were missed, 68 patients were on treatment, and six patients had no recorded sputum smear result as well as they were also on treatment. Because of the above-listed reasons we excluded from the analysis.  But at the revised manuscript based on the recommendations, you made before we have corrected as follows in the main document. “A total of 457 DR-TB patients who had baseline sputum smear result records were included to assess the impacts of diagnostic tests on treatment outcomes.”  To make clear how the number of patients comes to 457 we have added a diagram in the revised manuscript as **Figure 2.** |
| 6. Line 194, ‘six patients were on both’…. this is not clear when you state both treatments, which is which? | **Answer: Thank you, dear reviewer!**  Dear reviewer, We have addressed this question in question number 5 above. |
| 7. Table 4, need revision based on the recommended treatment outcome categorization (successful versus unsuccessful treatment outcome). See the previous comment in the methods section. | **Answer: Thank you, reviewer, for your comment.**  Dears, we have revised it according to your comments. **Table 4**, **Page: 14** |
| **Discussions** |  |
| Line 229-231, the sentence starting with ‘the rationale for this….’ This does not sound true. In fact, the opposite is true. The lower bacteria load delays time to culture positivity and the DST that follows culture. Please clarify or correct. | **Answer: Thank you, reviewer, for your comment.**  Dear reviewer, we have read your opinion and justification. We have tried to clear more in this regard in the main documents as follows. “This might be due to the required time to produce a result by each diagnosis method, the Xpert MTB/RIF assay will take two hours to produce results, LPA two days, and LJ culture will take weeks. On the contrary, we didn’t see a significant difference in the median time from the first care-seeking visit to the diagnosis and the first care-seeking visit to treatment among the three diagnostic tests of those patients who had baseline SS- results. These can be explained by the low MTB load in the SS- patients to be detected by an Xpert MTB/RIF test. The available Gene-Xpert machine in the study area was the conventional Gene-Xpert machine which requires hundreds of bacilli load/ml of sample. But, the LJ culture-based DST can grow and detect up to 10 live bacilli/ml of sample. Those patients who give sputum or other samples for LJ culture, the result will be available within three to six weeks. Whereas, in the study area if patients had a negative Xpert MTB/RIF test result because of low bacillary load or other reasons, they will be appointed to come and re-evaluated after three weeks of taking broad-spectrum antibiotics which may delay the diagnosis and treatment of DR-TB by three or more weeks. These three or more week delays in the Xpert MTB/RIF test and the normal taking by the solid culture median may produce a comparable median time between the two test methods.” **Page: 15 & 16, Line: 234-249.** |
| Line 232-237, this discussion is likely to change based on the comments in the methods and result on treatment outcomes (Table 4). Please revise it accordingly. | **Answer: Thank you, reviewer, for your comment.**  Dears, based on your suggestion we have re-analyze to see the effect of diagnostic tests on treatment outcome after recoding the treatment outcome in successful and unsuccessful and we found insignificant results, which is similar to the previous reports. Because of this, we kept the discussions unchanged. |
| Limitations: line 240, why was ‘the health care facilities’ included as missing data. Didn’t you know the four hospitals you studied? In fact, would have been best if you included the result on the comparison between the hospitals in median times and treatment outcomes. Also, the distribution of patients among the four hospitals is missing. Wiser if you include those. As a limitation, you also need to include the lack of data on socioeconomic status and mode of treatment whether DOTS, at facilities or community based, as some of the factors potentially affecting treatment outcomes. | **Answer: Thank you, dear reviewer, once again.**  Sorry for the lack of clarification. We have also added your recommendations as a limitation in the revised manuscript. We wrote the health facilities as missing data just to mean the detailed characteristics of each hospital service/setups like the availability and quality of medical care, nutritional supports, drug supplies, and so on. Dear Reviewer, the mode of treatment was health facilities based DOTs throughout the whole treatment period. We have already tried to show the distribution of patients among the four hospitals in **Table 1** of the main document. Regarding the effect of other factors on the final treatment outcome, there are several published reports on other factors potentially affecting the treatment outcome in the study area and it is also out of our objectives. **Page: 16, Line: 257-262.** |
| **Conclusions and recommendation** |  |
| Line 247…’particularly in smear-positive patients’, your discussion shows the opposite, and your data does not fully support this. Please revise. | **Answer: Thank you, reviewer, for raising this important concern.**  Dears, we have reviewed the result of our study and tried to improve the discussion part as seen from the main document. Disagreeing with you, the conclusion we made was based on our result you can see **Table 3**, which shows the Xpert MTB/RIF test significantly reduced the median time among smear-positive DR-TB as compared to the LPA and LJ culture-based diagnostic tests. The problem in the previous manuscript might be, we didn’t thoroughly discuss why this was happening. In the recent manuscript, we have revised the discussion related to the median time among smear-positive and negative DR-TB patients and tried to put the possible rational. **Page: 15 & 16, Line: 234-249.** |
| Line 247-248 may change after your reanalysis of treatment outcomes (Table 4). Please revise it accordingly. | **Answer: Thank you, dear reviewer, for your productive comments.**  Dear reviewer, as per your notes we have re-analyzed the outcome and the finding was similar to the earlier report, because of this we kept it as it is. |
| **Minor comments:** |  |
| **Abstract** |  |
| Line 27 and 28, delete ‘with p-value’ and ‘finally, the p-value was reported’. Mentioning these may not be that necessary. | **Answer: Thank you, reviewer, for your comment.**  Dears, we have removed it from the main manuscript. |
| **Introduction** |  |
| Line 54 and 55, tuberculosis is already abbreviated, thus use it as TB moving forward. There are lots of places in the manuscript like this, please correct accordingly. | **Answer: We thank you, dear reviewer, for your valuable suggestions.**  As a result of your suggestions, we have corrected it all over the main document. |
| Line 57, LPA need to be written in full since first-time use. | **Answer: Thank you, dear reviewer, for your comment.**  Convening to your comment we have written LPA in full as Line Probe Assay (LPA) in the manuscript. |
| Line 58, replace the sentence with…. ‘Shorten time to diagnosis, and reduced delay to treatment among DR-TB, and improved final treatment outcomes.’ | **Answer: Dear reviewer, we thank you for your c, valuable suggestion.**  According to your suggestion, we have substituted the sentence in the main document. |
| Line 62, **GeneXpert** is the instrument (or the platform) and **Xpert MTB/RIF** is the assay (test), please correct this throughout the manuscript. | **Answer: We gratefully thank you, dear reviewer, for your comment.**  Dears, We agree with your valuable comments and corrected it now throughout the main manuscript. |
| Line 90, replace ‘we are intended’ with ‘we aim’ | **Answer: Thank you very much, dear reviewer, for your comment.**  We take your comment and corrected it in the manuscript. |
| **Methods** |  |
| Line 105, add a comma after historically, | **Answer: Thank you, reviewer, for your comment.**  According to your suggestion, we have added the comma in the main document. |
| Line 116, replace the sentence in parenthesis with ‘data difference from the diagnosis to initiation of treatment.’ | **Answer: Thank you, reviewer, for your comment.**  Dears, we have corrected the main document as follows. “The date difference from the DR-TB diagnosis to initiation of anti-TB treatment”. |
| **Result** |  |
| Line 154, replace ‘three-hundred twenty-nine’ with 329 | **Answer: Thank you, reviewer, for your comment.**  We have replaced now |
| Line 155, remove ‘with’ after 28 and add old after ‘years’ | **Answer: Thank you, reviewer, for the comment.**  We have done accordingly in the main document. |
| Line 160, replace ‘drug-resistant tuberculosis’ with DR-TB. | **Answer: Dear reviewer thank you for your comment.**  We have corrected it in the main manuscript. |
| Line 161, replace ‘DR-TB diagnosis’ with ‘diagnostic’ | **Answer: Thank you, reviewer, for your comment.**  We have replaced it now in the main document. |
| Line 164-167, the use of semi-colon and comma does not flow well. Replace the sentences with *‘The overall median time and IQR in days from first care-seeking visit to diagnosis, from diagnosis to treatment, and from first care-seeking to treatment were: 2 (0,9), 3 (1,8) and 4 (1,11) for Xpert MTB/RIF; 4 (1,55), 33 (4,76) and 3 (1,12) for LPA; and 70 (18,182), 44 (9, 145) and 76 (3.75,191) for culture.’* …your numbers of course will be corrected per the revised analysis and result. | **Answer: Thank you very much, dear reviewer, for your valuable suggestions.**  According to your proposed, we have re-write it in the main documents. |
| Line 167, remove respectively. | **Answer: Thank you, reviewer, for the comment.**  Now, we have removed it from the main document |
| Line 194, use a text for a number in a single digit. Replace 6 with ‘six’ | **Answer: Dear reviewer thank you for your comment.**  We have replaced it |
| Line 194, add ‘s’ after the patient (patients) and replace was with were. Six patients were….. | **Answer: Thank you, reviewer, for your comment.**  We have corrected it accordingly in the main document. |
| **Discussion** |  |
| Line 212, replace ‘diagnosis TD-TB tool’ with ‘diagnostic tool’ | **Answer: Thank you, reviewer, for your comment.**  We have replaced it with the documents. |
| Line 216, replace GeneXpert with ‘Xpert MTB/RIF’ | **Answer: Thank you, dear reviewer, for your comment.**  We have replaced it throughout the documents. |
| Line 216, Xpert MTB/RIF test is not simple. It is a very complex process, but easy to perform. Thus replace ‘a simple diagnostic assay’ with ‘an easy to do diagnostic assay’.  Line 239, add ‘data’ after missing. | **Answer: Dear reviewer, thank you very much for your comment.**  We have corrected it in the main document as per the given comments. |
| **Acknowledgment** |  |
| Line 253, remove ‘s’ from datas… | **Answer: Thank you, reviewer, for your comment.**  We have removed it in the main document. |
| **Authors’ contribution** |  |
| Replace ‘Dta’ with ‘Data’. | **Answer: Thank you, reviewer, for your comment.**  We have replaced Dta with data in the main document. |
| **References** |  |
| Line 343, Ref # 24, seems not well written. Please revise. | **Answer: thank you, Dear reviewer.**  We have corrected it and incorporated it on the documents. |
| Line 363-4, the reference seems missing the publication number. Please revise. | **Answer: Thank you, Dear Reviewer.**  Now, we have added the publication number on the main documents. |
| It is good if authors include map of study area with indicated location of study hospitals on the map. | **Answer: Thank you, dear reviewer, once again.**  Accordingly, we have included a map of the study area. Figure 1. |
| Table titles are very long and needs to revise and make short and informative | **Answer: Thank you, Dear Reviewer.**  Accordingly, we have tried to shorten the titles of tables. **Table 1, 2, 3, & 4, page: 9, 11, 12 & 13.** |
| In discussion part: line 232-234. "There was no statistically significant difference in terms of final treatment outcomes in all of the DR-TB diagnostic algorisms. From literatures, we found a controversial result between the different diagnostic tools and treatment outcomes" The reason for this controversy might be confounding factor in your analysis and did author checked for confounding factors in there statistical analysis? | **Answer: Dear reviewer, thank you.**  As you have noted, these controversial findings were noticed from published papers, not from our findings. Some reports say there is a significant difference and some reports also said there is no. These might be because of different reasons like the confounding variables, methodological, and measurement issues. |
