# Supplementary material for: Comparing the impact of genotypic based diagnostic algorithm on time to treatment initiation and treatment outcomes among drug-resistant tuberculosis patients in Amhara region, Ethiopia
Source: PLoS One. 2021 Feb 18;16(2):e0246938. doi: 10.1371/journal.pone.0246938 (PMC7891731; doi:10.1371/journal.pone.0246938)
Supplement: Attachment — Submitted filename: Review PONE-D-20-18404_R1.docx [file pone.0246938.s003.docx]

**Review:** **‘Comparing the impact of Genotypic Based Diagnostic Algorithm on Time to Treatment Initiation and Treatment Outcomes among Drug-Resistant Tuberculosis Patients in Amhara Region, Ethiopia’.**

**PONE-D-20-18404_R1**

The topic under discussion remains very relevant and important to understand the effect of the molecular diagnostics in the early diagnosis and treatment initiation, and its impact on patient-level treatment outcome. In the revised form, the manuscript still needs major revision and correction in its methodology, analysis and discussion.

**Major comments:**

**Abstract**

The methods and the discussion section in the abstract need to be revised based on the detailed comments in the main manuscript. The major comments are around methods are clarifying how the three comparison categorizes were created.

**Introduction**

None

**Methods**

Comment *‘since the three tests are usually conducted one after another, it is not clear how the three distinctive comparison groups were categorized. For instance, if a sample tested positive by Xpert MTB/RIF with Rifampicin Resistance, then LPA needs to be conducted to confirm for Isoniazid resistance’*

The below response for the above comment needs to be summarized and included in the methods section. Without it the readers would still get confused in the process and the conclusion the authors attempted to reach.

*Xpert MTB/RIF was introduced in the Amhara region, Ethiopia by September 2013, before that culture and LPA was used alternatively to diagnose DR-TB. Even after the introduction of Xpert MTB/RIF in 2013, culture and LPA were also used to diagnose DR-TB because of the limited Xpert MTB/RIF test access. Currently, as you have stated these three tests are done one after the other. Once the patient becomes resistant to Rifampicin by Xpert MTB/RIF then first and second line LPA was performed to see further resistance for INH, FLQ, and injectable. The culture was done to monitor the treatment response and for further phenotypic DST performance.*

**Results**

1. Table 2, the median time from to diagnosis (33) and to treatment (3) discrepancies still need further discussion. The earlier is more the health system factor and the latter is more patient related factor such as delayed presentation for treatment after diagnosis. See comments on the discussion part. age in years…the 16.75, 28.5 and 17 are not IQR, this needs correction
2. Table 3, since the table is by smear status, either correct the heading to 540 or if you want to keep 574 include the median times for unknown smears too. Also, if you add 68,143 and 237 gives you 448, not 540 or 574. Please correct or clarify.

The result under LPA ss+ and ss-, 30 versus 48; and culture 84 versus 14 raised concern. E.g. one would not expect ss+ to have higher median time that ss- for obvious reasons. The higher the bacillary load the faster for the culture to convert to positive result, thus time to diagnosis should be lesser.

**Discussions**

**Line 231-232**, ‘*This was 232 supported by a study from Ethiopia [35]*’. How does ref #35 support you result? Rather, indicate the result how similar or different it is.

**Line 133-134**, *‘And we also observed that a significant delay in terms 233 of time from the first care-seeking visit to the diagnosis and the first care-seeking visit to 234 treatment among patients with baseline SSm+ result’*. This is only true for culture, and not indicated in your results. In addition, the comparison in your result (Table 3), is between diagnostics (culture, LPA and Xpert MTB/RIF), not ss+ versus ss-. To make your discussion appropriate you need the later analysis.

**Line 134-136**, again, the median time to diagnosis from first visit is more of a health system related factor and median time to treatment is more of a patient related factor such as delay to present to health facilities after diagnosis. Thus, you need to clarify and discuss the points in the detail and explanation they deserve.

**Line 136-139**, same comment as above applies, clarify your discussion points and make it more plausible. As it is lacks sufficient details.

**Line 242-249**, This discussion with ss-, you are contradicting yourself where your earlier points state ss+ has delays than ss-. Please revise and clarify your discussion here.

In addition, some discussion points are lacking as to why diagnosis to treatment in LPA is longer than first visit to treatment. LPA and Xpert has only one or two days difference in terms of turnaround time versus culture that may take months. Your clarification on skewedness does not fully explain.

Limitation, please include not presenting the DR-TB regimens as a limitation since one cannot discuss treatment outcome without describing the treatment regimen. It is even better, if you include in the methods section the clarification you provided in your response in this regard.

**Conclusions and recommendation**

Once you edited the discussion based on the comments provided you may revise the conclusion too.

**Minor comments:**

**Methods**

Figure 2, please edit the table to professional formatting. E.g. your arrows are not straight, not aligned and their sizes are not equal. Also, in the second box – replace ‘expert’ to Xpert MTB/RIF.

**Result**

Line 163, usually we use numbers when 10 and above. Replace fourteen with 14.
