# Supplementary material for: Comparing the impact of genotypic based diagnostic algorithm on time to treatment initiation and treatment outcomes among drug-resistant tuberculosis patients in Amhara region, Ethiopia
Source: PLoS One. 2021 Feb 18;16(2):e0246938. doi: 10.1371/journal.pone.0246938 (PMC7891731; doi:10.1371/journal.pone.0246938)
Supplement: Attachment — Submitted filename: Authors Response 2.docx [file pone.0246938.s004.docx]

**Date: January 14, 2021**

**Rebuttal letter**

PONE-D-20-18404R1

**Comparing the impact of Genotypic Based Diagnostic Algorithm on Time to Treatment Initiation and Treatment Outcomes among Drug-Resistant Tuberculosis Patients in Amhara Region, Ethiopia**

**Mehari Woldemariam Merid**

**To PLOS ONE**

**Dear all,**

We the authors of this manuscript are pleased to thank the journal editors and the reviewers for revising the manuscript and giving your valuable and constructive comments and suggestions that help to improve the manuscript. We have made a rigorous revision of the manuscript as per your questions and comments. We have included the point by point response in the table below framed as reviewers’ comment/question and authors’ response. The detailed revision and changes we made in the main document are prepared with track changes in the document attached separately. We expect that the revision we made will enable the manuscript to fit the journal. We are happy to receive additional revision if any that would have merit in improving the manuscript.

| **Reviewers comments** | **Authors Response** |
| --- | --- |
| **Major Comments** |  |
| **Abstract** |  |
| The methods and the discussion section in the abstract need to be revised based on the detailed comments in the main manuscript. The major comments are around methods are clarifying how the three comparison categorizes were created. | Dear reviwer, as per your sugestions we have revised the method parts of the abstract. Since there was no discussion part in the abstract we didn’t need to revise it. |
| **Methods** |  |
| Comment *‘since the three tests are usually conducted one after another, it is not clear how the three distinctive comparison groups were categorized. For instance, if a sample tested positive by Xpert MTB/RIF with Rifampicin Resistance, then LPA needs to be conducted to confirm for Isoniazid resistance’*  The below response for the above comment needs to be summarized and included in the methods section. Without it the readers would still get confused in the process and the conclusion the authors attempted to reach.  *Xpert MTB/RIF was introduced in the Amhara region, Ethiopia by September 2013, before that culture and LPA was used alternatively to diagnose DR-TB. Even after the introduction of Xpert MTB/RIF in 2013, culture and LPA were also used to diagnose DR-TB because of the limited Xpert MTB/RIF test access. Currently, as you have stated these three tests are done one after the other. Once the patient becomes resistant to Rifampicin by Xpert MTB/RIF then first and second line LPA was performed to see further resistance for INH, FLQ, and injectable. The culture was done to monitor the treatment response and for further phenotypic DST performance.* | **Thanks, Dear Reviewer**  We had summarized and incorporated it into the method parts of the manuscript as follows. “Culture-based DST and LPA was used alternatively to diagnose DR-TB in Ethiopia until 2013. Latter in 2013, Xpert MTB/RIF was introduced in the study area to diagnose RR-TB. Currently, only TB culture-based DST and LPA sites are available in the Amhara region, which is located in Bahr-Dar and Gondar. The access of these three diagnosis tests varies from site to site, because of this and some other reasons even after the introduction of Xpert MTB/RIF, culture and LPA were also used as the first test to diagnose DR-TB. Currently, those three tests are done serially. Once the patient was diagnosed to have RR-TB by Xpert MTB/RIF then first and the second line LPA was performed to see further resistance for Isoniazid, Floroqunolols, and injectable ant-TB drugs, and culture was done to monitor the treatment response and when further phenotypic DST is necessary.” |
| **Results** |  |
| 1. Table 2, the median time from to diagnosis (33) and to treatment (3) discrepancies still need further discussion. The earlier is more the health system factor and the latter is more patient related factor such as delayed presentation for treatment after diagnosis. See comments on the discussion part. age in years…the 16.75, 28.5 and 17 are not IQR, this needs correction | Sure, there were some patients presented with advanced and debilitating TB disease and highly indexed for DR-TB. In that case, the clinical panel team at the DR-TB center may start the standard DR-TB treatment clinically for those patients when the laboratory result is pending and revised their decision when the laboratory result was available.  We didn’t get the point of what you want to raise related to age. There was no age in table two. Age was described in table one as median and IQR. |
| 2. Table 3, since the table is by smear status, either correct the heading to 540 or if you want to keep 574 include the median times for unknown smears too. Also, if you add 68,143 and 237 gives you 448, not 540 or 574. Please correct or clarify. | Dear Reviewer, you are right the sum of 68,143 and 237 gives 448, not 540 or 574. This is a typo error. The error was, we wrongly writing only the number of SS+ (448) patients diagnostic in each tests, now we have corrected it by adding 92 SSm- DR-TB patients. |
| 3. The result under LPA ss+ and ss-, 30 versus 48; and culture 84 versus 14 raised concern. E.g. one would not expect ss+ to have higher median time that ss- for obvious reasons. The higher the bacillary load the faster for the culture to convert to positive result, thus time to diagnosis should be lesser. | The result under LPA doesn’t raise concern because the time in SS+ 30 days was shorter than SS- 48 days.  As you have stated there might be concerns on culture-based DSTs, for ss+ 84 days verses for ss- 14 days. This might be due to, at the first two years year of the DR-TB diagnosis and treatment history, the diagnosis was made from high-risk patients like patients with smear-positive by referring the sample to the Ethiopian public health institute located in Addis Abeba. At this time there may delays due to transportation, late reporting results. |
| **Discussions** |  |
| **Line 231-232**, ‘*This was 232 supported by a study from Ethiopia [35]*’. How does ref #35 support you result? Rather, indicate the result how similar or different it is. | Dear Reviewer, we have corrected it in the main manuscript based on your suggestions as follows. “Similar result was reported from another study in Ethiopia (35).” |
| **Line 133-134**, *‘And we also observed that a significant delay in terms 233 of time from the first care-seeking visit to the diagnosis and the first care-seeking visit to 234 treatment among patients with baseline SSm+ result’*. This is only true for culture, and not indicated in your results. In addition, the comparison in your result (Table 3), is between diagnostics (culture, LPA and Xpert MTB/RIF), not ss+ versus ss-. To make your discussion appropriate you need the later analysis. | Dear reviewer, the problem we have observed here is our poor sentence construction, which doesn’t communicate the message about what we want to forward. We have tried to clarify the sentence as follows in the main document. “And we also observed that a significant median time deference from the first care-seeking visit to the diagnosis, the diagnosis to treatment, and the first care-seeking visit to treatment in the three DR-TB diagnostic tests among patients with baseline SSm+ (Table 3).” |
| **Line 134-136**, again, the median time to diagnosis from first visit is more of a health system related factor and median time to treatment is more of a patient related factor such as delay to present to health facilities after diagnosis. Thus, you need to clarify and discuss the points in the detail and explanation they deserve. | Dear reviewer, we accept your suggestions and had discussed it as follows. “This might be due to the required time to produce a result by each diagnosis method, the Xpert MTB/RIF assay will take two hours to produce results, LPA two days, and LJ culture will take weeks. It can be also due to a health facility and patient-related delays. One, there were several DR-TB patients on the waiting list to start second-line anti-TB drugs after their DR-TB diagnosis because of a shortage of beds and drugs. Two, DR-TB diagnostic tests were not available onsite to all of the DR-TB TICs in the region. Because of this, samples were collected from patients and stored in a refrigerator till transported to the sample processing sites. The first DR-TB diagnostic site in Ethiopia was the Ethiopian Public Health Institute (EPHI) located in Addis Abeba (the capital city of Ethiopia) followed by the Amhara Public Health Institute (APHI) located in Bahr-Dar, which is too far from these TICs. The sample from the patients at each TICs was collected, stored, and send to EPHI or APHI for further diagnostic tests. The days taken for sample storage, transportation, and late report of results may also produce dalliance in the diagnosis and treatment of patients. Three, the patient may delay presenting to health facilities after diagnosis.” |
| **Line 136-139**, same comment as above applies, clarify your discussion points and make it more plausible. As it is lacks sufficient details. |  |
| **Line 242-249**, This discussion with ss-, you are contradicting yourself where your earlier points state ss+ has delays than ss-. Please revise and clarify your discussion here. | Dear reviewer, we agree with you, this contradicting discussion was established because of the poor sentence construction in the SSm+. Now we have re-write the sentence to make the discussion clear in the main manuscript. |
| In addition, some discussion points are lacking as to why diagnosis to treatment in LPA is longer than first visit to treatment. LPA and Xpert has only one or two days difference in terms of turnaround time versus culture that may take months. Your clarification on skewedness does not fully explain. | Sure, these were because of the accessibility of tests and there was also a platform for rapid reporting of results for Xpert MTB/RIF tests. |
| Limitation, please include not presenting the DR-TB regimens as a limitation since one cannot discuss treatment outcome without describing the treatment regimen. It is even better, if you include in the methods section the clarification you provided in your response in this regard. | Thank you, dear reviewer, we had included in the limitation that this study lacks to assess the effect of DR-TB regimens on final treatment outcomes in the main manuscript. |
| **Conclusions and recommendation** |  |
| Once you edited the discussion based on the comments provided you may revise the conclusion too. | Dear reviewer, we dint find reasons that can enable us to revise our conclusion. |
| **Minor comments:** |  |
| **Methods** |  |
| Figure 2, please edit the table to professional formatting. E.g. your arrows are not straight, not aligned and their sizes are not equal. Also, in the second box – replace ‘expert’ to Xpert MTB/RIF. | Thank you, dear reviewer, we have edited figure two according to your comments. |
| **Result** |  |
| Line 163, usually we use numbers when 10 and above. Replace fourteen with 14. | Thank you dears, we have replaced it. |
